# An In Vivo Study on Wound Healing Activity of *Prangos* Species and Phytochemical Investigations

**DOI:** 10.3390/molecules31213974

**Published:** 2026-11-04

**Authors:** Nazif Gülmez, Alev Onder, İpek Süntar, Bayram Kagan Akay, Mürşide Ayşe Demirel, Songül Çeribaşı, Erkan Yilmaz

**Affiliations:** 1 Department of Pharmacognosy, Faculty of Pharmacy, Ankara University, Ankara 06560, Türkiye; 2 Graduate School of Health Sciences, Ankara University, Dışkapı, Ankara 06110, Türkiye; 3 Department of Pharmacognosy, Faculty of Pharmacy, Gazi University, Etiler, Ankara 06330, Türkiye; 4 Department of Pharmaceutical Basic Sciences, Faculty of Pharmacy, Gazi University, Ankara 06630, Türkiye; aysedemirel@gazi.edu.tr; 5 Department of Pathology, Faculty of Veterinary Medicine, Fırat University, Elazığ 23119, Türkiye; 6 Department of Pharmacognosy, Faculty of Pharmacy, Adıyaman University, Adıyaman 02040, Türkiye; erkanyilmaz@adiyaman.edu.tr

**Keywords:** *Prangos*, *Prangos denticulata*, aerial part, coumarins, wound-healing, ethnopharmacology

## Abstract

Wound healing is a dynamic and highly coordinated biological process involving four overlapping phases, as hemostasis, inflammation, proliferation, and tissue remodeling. The genus *Prangos* is known as “Çakşır” in Turkish and is an ethnomedicinal plant traditionally used in Anatolia for many purposes, including wound healing. The plant species is rich in furanocoumarins and their derivatives. This study aimed to investigate the wound-healing activity of selected endemic *Prangos* species, which are frequently mentioned in traditional medicine. For this purpose, several endemic species, such as *Prangos abieticola* Aytaç & H. Duman, *Prangos denticulata* Fisch. & C.A. Mey., *Prangos heyniae* H. Duman & M.F. Watson, and *Prangos uechtritzii* Boiss. & Hausskn. were collected and extracted for comparison of in vivo wound-healing activities. We evaluated the wound-healing activity of methanol extracts of the aerial parts of these species using a circular excision wound model. Tissue levels of TGF-*β*, hydroxyproline, total antioxidant status (TAS), total oxidant status (TOS), and oxidative stress index (OSI) were measured. The methanol extract of *P. denticulata* exhibited notable wound-healing activity. Therefore, in this study, *P. denticulata* was selected as a candidate for phytochemical investigations, and the methanol extract of the species was subjected to a successive chromatographic process. Phytochemical studies yielded seven known furanocoumarin-type compounds (**1–9**): isoimperatorin (**1**), imperatorin (**2**), oxypeucedanin methanolate (**3**), xanthotoxol (**4**), isogospherol (**5**), saxalin (**6**), and isosaxalin (**7**), along with a simple coumarin, ulopterol (**8**), and a linear-type dihydropyranocoumarin called decursinol angelate/agasyllin (**9**). The structures were elucidated using ^1^H, ^13^C, and 2D NMR spectroscopy, as well as MS fragmentation. This study reports the wound-healing activities of endemic species growing in Anatolian flora, along with the phytochemical content of the active species, *P. denticulata*. Since coumarin-type compounds are specific and dominant in *Prangos* species, it is considered that the wound-healing activity could be attributed to these compounds.

## 1. Introduction

Medicinal plants are natural resources used in various cultures for thousands of years to prevent, treat, and promote health [1]. Investigations of medicinal plants and the discovery of drug molecules provide important information about multifaceted and diverse pharmacological targets [2]. Apiaceae (formerly Umbelliferae) is a group of medicinal and aromatic plants that is considered one of the most important families of flowering plants worldwide [3]. The genus *Prangos* (Apiaceae) grows in the Irano–Turanian phytogeographical region. Most endemic species occur in the high mountainous areas of this region [4,5]. The genus *Prangos* Lindl. is represented by 40–50 taxa worldwide, whereas 21 taxa (13 of which are endemic) have been recorded in Türkiye [6,7,8].

The genus *Prangos* comprises medicinal and aromatic species that have long been utilized in traditional medicine for a variety of therapeutic purposes, particularly for wound healing and reputed aphrodisiac properties [9,10,11]. *P. pabularia* [12,13,14,15,16] and *P. platychlaena* [13,17,18,19] are some of the mentioned species for wound-healing effects in traditional medicine. The extract of *P. platychlaena* has been noted for its wound-healing effects in traditional use, treating abscesses, cuts, infected wounds, skin diseases, warts, and promoting wound-healing [17], and is recorded to be used in folk medicine in Eastern Anatolia to treat wounds under the tails of cattle [18]. *P. pabularia*, known as “Yugan” in Tajikistan, is reported to be used in the treatment of leukoplakia and scars [20]. Moreover, *P. ferulacea* and *P. pabularia* are used as a stimulant in the form of a decoction and are known as “Çaşır/Çakşır” in East Anatolia [21]. The infusion from aerial parts of *P. asperula* is used in wound infections [22,23]. The root of *P. ferulacea* is also treated in traditional medicine as a wound-healing agent (in the form of a topical ointment) for purulent wounds in humans and animals, particularly in Iran, based on knowledge from the literature and local people [24]. Similarly, the species is used for wound-healing purposes in Iran, prepared as a poultice and applied topically as a compress [25].

Phytochemical studies have revealed that these species are a rich source of secondary metabolites with significant biological activity, particularly furanocoumarins and simple coumarins [9,10].

Wounds, resulting from trauma, burns, surgical procedures, or underlying pathological conditions, remain a major global healthcare challenge due to their high prevalence, prolonged treatment, and substantial economic burden [26,27]. It is characterized by disruption of the skin’s epithelial integrity, leading to structural and functional damage that may range from superficial lesions to deep injuries involving multiple tissue layers [28]. Restoration of tissue integrity is achieved through wound healing, a tightly regulated biological process comprising four overlapping phases: hemostasis, inflammation, proliferation, and tissue remodeling [29]. Successful healing depends on the coordinated interplay of numerous cellular and molecular events, including inflammatory mediator release, cytokine signaling, oxidative stress regulation, angiogenesis, extracellular matrix formation, and collagen deposition [30,31].

Given the complexity of the wound healing process and the limitations associated with conventional therapies, there is growing interest in medicinal plants as complementary therapeutic agents. Among these, the *Prangos* species have attracted attention because of their rich phytochemical composition and long-standing use in traditional medicine. Considering these issues, the endemic *Prangos* species were compared with each other regarding this activity. Although various ethnopharmacological studies have been conducted on the traditional uses of *Prangos* species, the number of studies that scientifically prove these traditional uses is quite limited. In this study, we selected four endemic species belonging to the genus *Prangos* to evaluate the in vivo wound-healing activity of extracts prepared from the aerial parts using methanol. Linear incision and circular excision wound models and hydroxyproline determination were used to investigate the wound-healing activity. Moreover, the phytochemical profile of the active species, *Prangos denticulata*, was investigated for the first time, and its possible relationship with this activity was also evaluated.

## 2. Results

### 2.1. Extraction and Isolation

The samples from different *Prangos* species (*P. abieticola* Aytaç & H.Duman, *P. denticulata* Fisch. & C.A.Mey., *P. heyniae* H.Duman & M.F.Watson, and *P. uechtritzii* Boiss. & Hausskn.) were used for the activity tests.

Based on the activity test results from ongoing studies, a larger quantity of plant powder was used to extract the active compounds. For this, the air-dried and powdered aerial parts of *P. denticulata* (500 g) were extracted with methanol using a Soxhlet apparatus (10 extraction cycles) after being determined as an active species. The combined extracts were concentrated under reduced pressure to afford a crude methanolic extract (90 g/dry extract) and kept in a refrigerator until the experiments.

The isolation and purification process were carried out using general chromatographic techniques. A portion of the crude extract (80 g) was subjected to vacuum-assisted column chromatography over silica gel. The extract was coarsely fractionated to yield eight major fractions (Fr. 1–8) eluting successively with hexane, ethyl acetate, and methanol. Fraction analysis was performed based on thin-layer chromatography (TLC) on silica gel 60F_254_ plates developed with different polarities of hexane–ethyl acetate solvent systems. Final purification of the constituents was achieved by recrystallization (compound **1**, isoimperatorin) from fractions 2–4 (9 g), repeated column chromatography, and preparative TLC, as appropriate. Following phytochemical investigation of fractions 2–4, and 5 (2 g) eluting with of *n*-hexane: EtOAc gradient systems resulted in the isolation of nine known coumarins: isoimperatorin (**1**, 20 mg), imperatorin (**2**, 10 mg), oxypeucedanin methanolate (**3**, 15 mg), xanthotoxol (**4**, 4 mg), isogosferol (**5**, 6 mg), saxalin (**6**, 3 mg), isosaxalin (**7**, 10 mg), ulopterol (**8**, 10 mg), and decursinol angelate (**9**, 15 mg). At the end, total isolation results showed that fraction 2 afforded isoimperatorin (**1**), and imperatorin (**2**), whereas fraction 3 yielded oxypeucedanin methanolate (**3**) and xanthotoxol (**4**). Further purification of fractions 5 and 6 resulted in the isolation of isogosferol (**5**), saxalin (**6**), isosaxalin (**7**), ulopterol (**8**), and decursinol angelate (**9**).

*Isoimperatorin* (**1**)

White needles, C_16_H_14_O_4_: *m/z* 270, *m/z* 293 [M+Na]^+^, ^1^H NMR (500 MHz, CDCl_3_): *δ* 6.26 (1 H, d, *J* = 9.8, H-3), 8.15 (1 H, d, *J* = 9.8, H-4), 7.14 (1 H, s, H-8), 7.59 (1 H, d, *J* = 2.4, H-2′), 6.95 (1 H, d, *J* = 0.8, 2.4, H-3′), 4.92 (2 H, d, *J* = 7.0, H-1″), 5.53 (1 H, tp, *J* = 7.0, 1.35, H-2″), 1.80 (3 H, s, H-4″), 1.70 (3 H, s, H-5″). ^13^C NMR (125 MHz, CDCl_3_): *δ* 161.3 (C-2), 112.6 (C-3), 139.6 (C-4), 107.5 (C-4a), 148.9 (C-5), 114.2 (C-6), 158.1 (C-7), 94.2 (C-8), 152.7 (C-8a), 144.9 (C-2′), 105.0 (C-3′), 69.7 (C-1″), 119.0 (C-2″), 139.9 (C-3″), 18.2 (C-4″), 25.8 (C-5″).

*Imperatorin* (**2**)

Needles, C_16_H_14_O_4_: *m/z* 270, *m/z* 293 [M+Na]^+^, ^1^H NMR (500 MHz, CDCl_3_): *δ* 6.35 (1 H, d, *J* = 9.7, H-3), 7.75 (1 H, d, *J* = 9.7, H-4), 7.34 (1 H, s, H-5), 7.67 (1 H, d, *J* = 2.1, H-2′), 6.80 (1 H, d, *J* = 2.1, H-3′), 4.99 (2 H, d, *J* = 7.0, H-1″), 5.60 (1 H, tp, *J* = 7.0, 1.3, H-2″), 1.72 (3 H, s, H-4″), 1.70 (3 H, s, H-5″). ^13^C NMR (125 MHz, CDCl_3_): *δ* 160.7 (C-2), 114.6 (C-3), 144.5 (C-4), 116.5 (C-4a), 113.2 (C-5), 125.9 (C-6), 148.6 (C-7), 131.6 (C-8), 143.8 (C-8a), 146.7 (C-2′), 106.7 (C-3′), 70.6 (C-1″), 119.7 (C-2″), 139.8 (C-3″), 18.1 (C-4″), 25.8 (C-5″).

*Oxypeucedanin methanolate* (**3**)

Amorph powder, C_17_H_18_O_6_: *m/z* 318, *m/z* 341 [M+Na]^+^, ^1^H NMR (500 MHz, CDCl_3_): *δ* 6.28 (1 H, d, *J* = 9.6, H-3), 8.22 (1 H, d, *J* = 9.6, H-4), 7.16 (1 H, s, H-8), 7.59 (1 H, dd, *J* = 1.3, 0.8, H-2′), 7.00 (1 H, dd, *J* = 1.3, 0.8, H-3′), 4.38 (1 H, dd, *J* = 7.9, 9.9, H-1″a), 4.56 (1 H, dd, *J* = 2.9, 9.9, H-1″b), 3.94 (1 H, dd, *J* = 2.9, 7.9, H-2″), 3.26 (3 H, s, -OCH3), 1.24 (3 H, s), 1.23 (3 H, s). ^13^C NMR (125 MHz, CDCl3): *δ* 161.3 (C-2), 112.8 (C-3), 139.4 (C-4), 107.3 (C-4a), 148.8 (C-5), 114.0 (C-6), 148.1 (C-7), 94.5 (C-8), 152.6 (C-8a), 145.0 (C-2′), 104.9 (C-3′), 74.3 (C-1″), 76.1 (C-2″), 75.9 (C-3″), 49.3 (C-3″ -OCH3), 20.8 (C-4″), 20.7 (C-5″).

*Xanthotoxol* (**4**)

Amorph, C_11_H_6_O_4_: *m/z* 202, *m/z* 225 [M+Na]^+^, ^1^H NMR (500 MHz, CDCl3): *δ* 6.40 (1 H, d, *J* = 9.6, H-3), 7.83 (1 H, d, *J* = 9.6, H-4), 7.30 (1 H, s, H-5), 7.74 (1 H, d, *J* = 2.0, H-2′), 6.84 (1 H, d, *J* = 2.0, H-3′). ^13^C NMR (125 MHz, CDCl_3_): *δ* 160.2 (C-2), 114.3 (C-3), 144.9 (C-4), 115.8 (C-4a), 110.6 (C-5), 126.1 (C-6), 144.4 (C-7), 129.4 (C-8), 138.9 (C-8a), 147.1 (C-2′), 106.8 (C-3′).

*Isogospherol* (**5**)

Amorph, C_16_H_14_O_5_: *m/z* 286, *m/z* 309 [M+Na]^+^, ^1^H NMR (500 MHz, CDCl_3_): δ 6.41 (1 H, d, *J* = 9.6, H-3), 7.80 (1 H, d, *J* = 9.6, H-4), 7.43 (1 H, s, H-5), 7.73 (1 H, d, *J* = 2.2, H-2′), 6.86 (1 H, d, *J* = 2.2, H-3′), 4.33 (1 H, dd, *J* = 8.5, 10.1, H-1″a), 4.65 (1 H, dd, J = 10.0, 2.9 H-1″b), 4.57 (1 H, dd, J = 2.5, 8.5, H-2″), 5.19 (1 H, brs, H-4″a), 5.02 (1 H brm, *J* = 0.6, 4″b), 1.85 (3 H, brs, H-5″). ^13^C NMR (125 MHz, CDCl*3*): *δ* 160.2 (C-2), 114.9 (C-3), 144.3 (C-4), 116.6 (C-4a), 113.9 (C-5), 125.9 (C-6), 148.2 (C-7), 131.6 (C-8), 142.7 (C-8a), 146.8 (C-2′), 106.9 (C-3′), 77.4 (C-1″), 73.9 (C-2″), 143.6 (C-3″), 112.9 (C-4″), 18.1 (C-4″), 18.9 (C-5″).

*Saxalin* (**6**)

White needles, C_16_H_15_O_5_Cl: *m/z* 322, *m/z* 323 [M+H]^+^, *m/z* 345 [M+Na]^+^, ^1^H NMR (500 MHz, CDCl_3_): *δ* 6.30 (1 H, d, *J* = 9.7, H-3), 8.19 (1 H, d, *J* = 9.7, H-4), 7.19 (1 H, s, H-8), 7.62 (1 H, d, *J* = 2.2, H-2′), 6.99 (1 H, d, *J* = 2.2, H-3′), 4.71 (1 H, dd, *J* = 3.1, 10.1, H-1″), 4.46 (1 H, dd, *J* = 7.5, 10.0, H-2″), 4.06 (1 H, dd, *J* = 8.5, 2.5), 1.70 (3 H, s, H-4″ and H-5″). ^13^C NMR (125 MHz, CDCl_3_): *δ* 161.1 (C-2), 113.2 (C-3), 139.0 (C-4), 107.4 (C-4a), 148.3 (C-5), 114.2 (C-6), 158.1 (C-7), 94.9 (C-8), 152.6 (C-8a), 145.4 (C-2′), 104.7 (C-3′), 74.1 (C-1″), 77.4 (C-2″), 71.5 (C-3″), 28.7 (C-4″), 29.0 (C-5″).

*Isosaxalin* (**7**)

Amorph, C_16_H_15_O_5_Cl: *m/z* 322, *m/z* 345 [M+Na]^+^, ^1^H NMR (500 MHz, CDCl_3_): *δ* 6.40 (1 H, d, *J* = 9.6, H-3), 7.79 (1 H, d, *J* = 9.6, H-4), 7.42 (1 H, s, H-5), 7.73 (1 H, d, *J* = 2.2, H-2′), 6.86 (1 H, d, *J* = 2.2, H-3′), 4.89 (1 H, dd, *J* = 3.0, 10.2, H-1″), 4.48 (1 H, dd, *J* = 8.0, 10.2, H-2″), 4.12 (1 H, dd, *J* = 3.0, 8.0), 1.70 (3 H, s, H-4″), 1.72 (3 H, s, H-5″). ^13^C NMR (125 MHz, CDCl_3_): *δ* 160.2 (C-2), 114.9 (C-3), 144.3 (C-4), 116.5 (C-4a), 113.9 (C-5), 126.1 (C-6), 148.0 (C-7), 131.6 (C-8), 143.4 (C-8a), 146.9 (C-2′), 106.9 (C-3′), 75.2 (C-1″), 77.2 (C-2″), 70.9 (C-3″), 28.4 (C-4″), 29.6 (C-5″).

*Ulopterol* (**8**)

Amorph, C_15_H_18_O_5_: *m/z* 278, *m/z* 301 [M+Na]^+^, ^1^H NMR (500 MHz, CDCl_3_): *δ* 6.24 (1 H, d, *J* = 9.5, H-3), 7.62 (1 H, d, *J* = 9.5, H-4), 7.31 (1 H, s, H-5), 6.80 (1 H, s, H-8), 3.89 (3 H, s, H-7, -OCH3), 2.99 (1 H, dd, *J* = 2.0, 13.5, H-1″a), 2.53 (1 H, dd, *J* = 10.5, 13.5, H-1″b), 3.63 (1 H, dd, *J* = 2.0, 10.5, H-2″), 1.31 (3 H, s, H-4″), 1.27 (3 H, s, H-5″). ^13^C NMR (125 MHz, CDCl_3_): *δ* 160.7 (C-2), 113.1 (C-3), 143.5 (C-4), 129.6 (C-4a), 124.9 (C-5), 161.4 (C-6), 98.9 (C-7), 56.0 (C-7, -OCH3), 154.6 (C-8), 112.1 (C-8a), 32.6 (C-1′), 77.6 (C-2′), 73.0 (C-3″), 26.4 (C-4′), 23.5 (C-5′).

*Decursinol angelate/Agasyllin* (**9**)

Amorph, C_19_H_20_O_5_: *m/z* 328, *m/z* 351 [M+Na]^+^, ^1^H NMR (500 MHz, CDCl^3^): *δ* 6.21 (1 H, d, *J* = 9.5, H-3), 7.60 (1 H, d, *J* = 9.5, H-4), 7.22 (1 H, s, H-5), 6.73 (1 H, s, H-8), 3.26 (2 H, m, H-1′), 5.06 (1 H, dd, *J* = 7.6, 9.3, H-2′), 1.62 (3 H, s, H-4′), 1.61 (3 H, s, H-5′), 5.98 (1 H, qq, J = 7.2, 1.4, H-1″), 1.67 (3 H, p, *J* = 1.5, H-4″), 1.89 (3 H, dq, *J* = 7.2, 1.5, H-5″). ^13^C NMR (125 MHz, CDCl_3_): *δ* 161.5 (C-2), 112.2 (C-3), 143.7 (C-4), 112.6 (C-4a), 123.2 (C-5), 124.5 (C-6), 163.4 (C-7), 97.8 (C-8), 155.7 (C-8a), 29.6 (C-1′), 89.1 (C-2′), 82.0 (C-3′), 22.3 (C-4′), 21.4 (C-5′), 167.1 (C-1″), 128.7 (C-2″), 137.7 (C-3″), 20.4 (C-4″), 15.6 (C-5″).

### 2.2. Wound Healing-Related Parameters and Oxidative Stress

**Figure 1:**
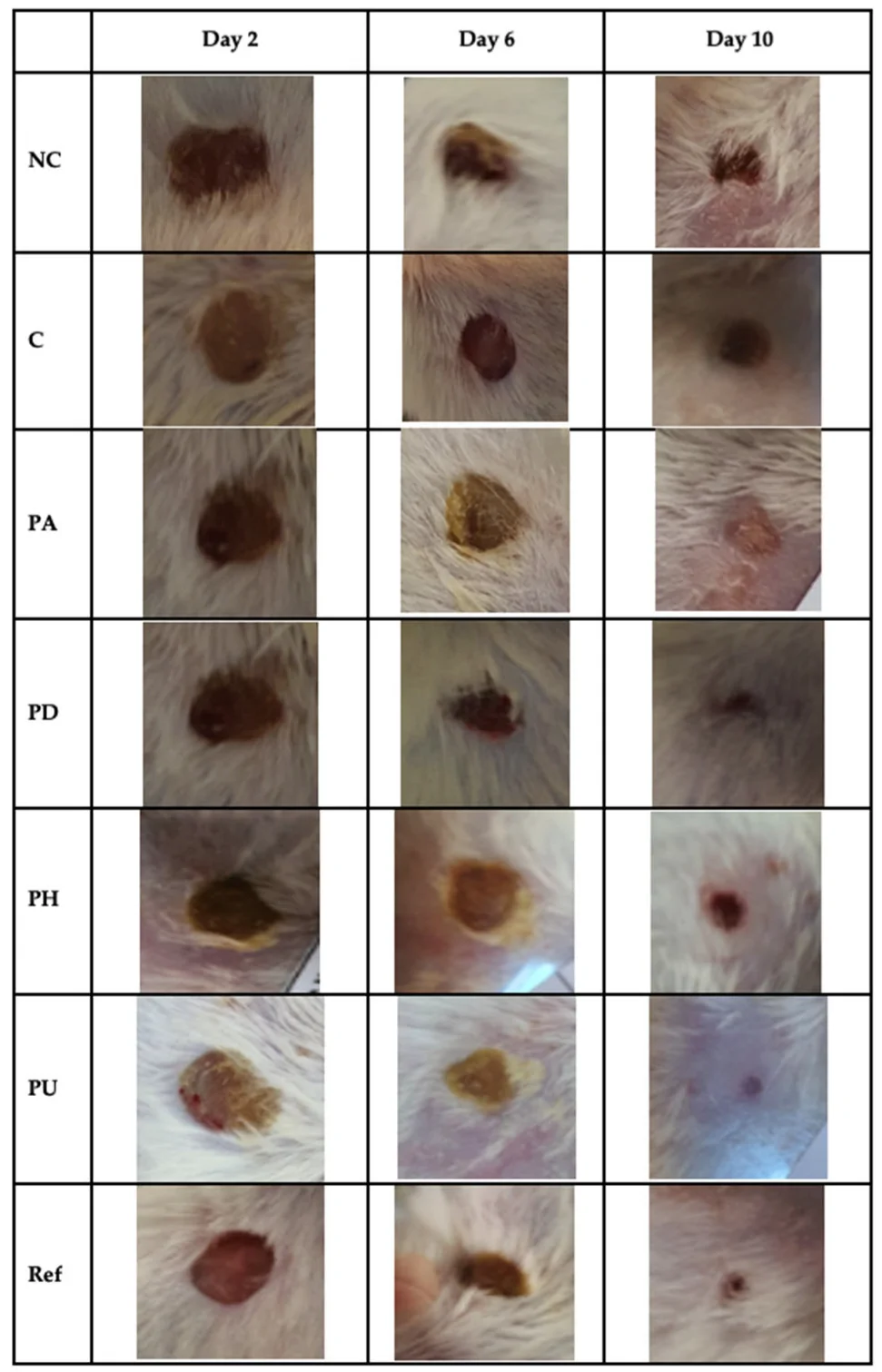
Macroscopic views of wound areas of mice treated with extracts of different *Prangos* species. C: Negative control; C: Control (Vehicle ointment); PA: *Prangos abieticola;* PD: *Prangos denticulata;* PH: *Prangos heyniae;* PU: *Prangos uechtritzii*; Ref: Reference drug (nitrofuzaone).

The wound area measurements were compared with those of the control (C) group. As a result of the comparison, it was determined that *P. denticulata* was the species that provided the most healing in wound areas (Table 1 and Figure 1).

On day 5, the tissue levels of TGF-*β* in the PA group were significantly higher than those in the C group (Table 2).

At the end of the 10th day, hydroxyproline levels in the tissues were found to be significantly higher in the PH group than in the C group. In the TAS measurements, the values of PD and the reference group were found to be significantly higher than those of the C group (Table 3).

### 2.3. Histological Analysis

The wound healing scores according to microscopic examination in the control and experimental groups are summarized in Table 4. At the end of 10 days, a statistically significant difference was found between the control and experimental groups in terms of granulation tissue and inflammatory infiltration parameters (*p* ˂ 0.05). However, no significant differences were observed in re-epithelialization, collagen deposition, angiogenesis, or ulcer formation (Table 4).

**Figure 2:**
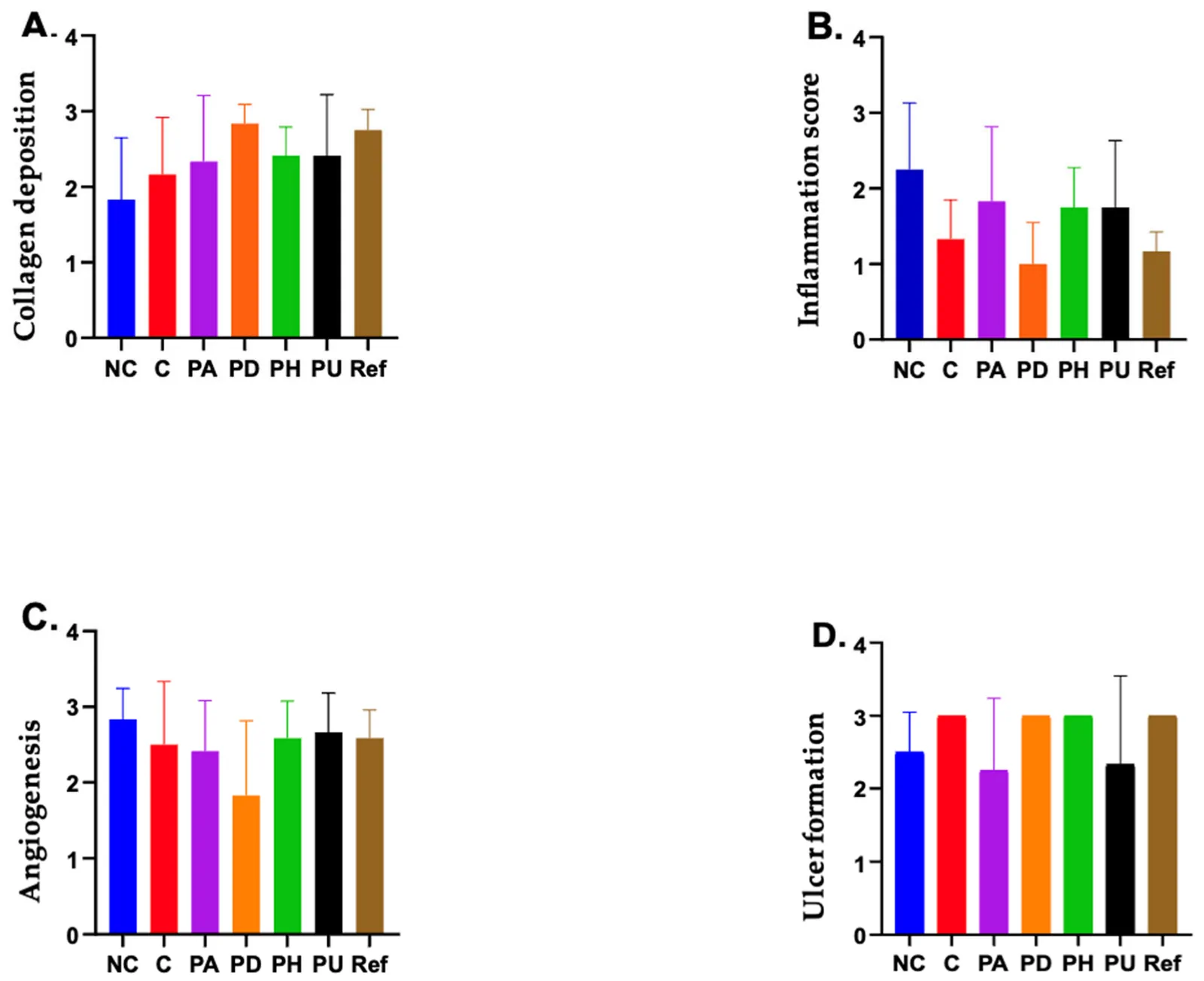
Histopathological evaluation of wound healing in mice treated with extracts of different *Prangos* species. (**A**). Collagen deposition, (**B**). Severity of inflammatory cell infiltration, (**C**). Angiogenesis, (**D**). Ulcer formation. NC: Negative control; C: Control (Vehicle ointment); PA: *Prangos abieticola*; PD: *Prangos denticulata*; PH: *Prangos heyniae*; PU: *Prangos uechtritzii*; Ref: Reference drug (nitrofuzaone).

**Figure 3:**
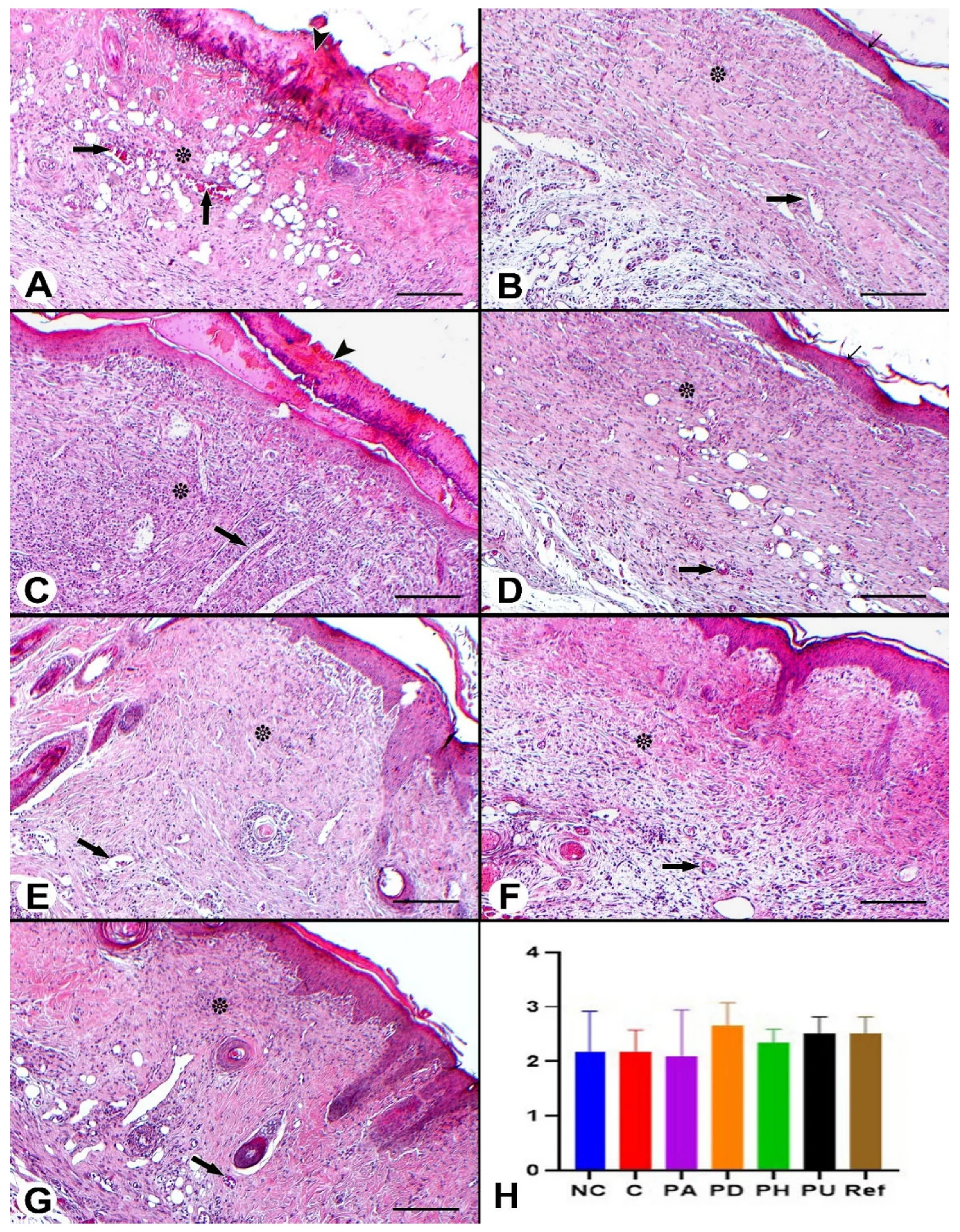
Histopathological evaluation of excisional wound healing in mice treated with extracts of different *Prangos* species. Representative hematoxylin and eosin (H&E)-stained sections of wound tissues from the experimental groups are shown. (**A**) NC group: absence of re-epithelialization beneath the scab (arrowhead), a small amount of granulation tissue (asterisk), and marked angiogenesis (large arrow). (**B**) C group: complete and mature epithelialization (thin arrow), mature granulation tissue (asterisk), and minimal angiogenesis (thick arrow). (**C**) PA group: complete but immature epithelialization beneath the scab (arrowhead), granulation tissue with prominent cellular infiltration (asterisk), and angiogenesis (arrow). (**D**) PD group: complete and mature epithelialization (thin arrow), mature granulation tissue (asterisk), and angiogenesis (large arrow). (**E**) PH group: mature granulation tissue (asterisk) and angiogenesis (large arrow). (**F**) PU group: granulation tissue (asterisk) with prominent cellular infiltration and capillary vessels (large arrow). (**G**) Ref group: mature granulation tissue (asterisk) and angiogenesis (large arrow). (**H**) Quantitative evaluation of re-epithelialization in the control and experimental groups. H&E staining, ×50 magnification; scale bar = 100 µm. NC: Negative control; C: Control (Vehicle ointment); PA: *Prangos abieticola;* PD: *Prangos denticulata;* PH: *Prangos heyniae*; PU: *Prangos uechtritzii*; Ref: Reference drug (nitrofuzaone).

Re-epithelialization was best in the PD, PU, and Reference groups. Less successful epithelialization occurred in the NC, Control, and PA groups than in the aforementioned groups (Figure 2A–D). When the groups were considered in terms of granulation tissue formation, it was observed that it occurred most prominently in the PD group (Figure 3A–G).

**Figure 4:**
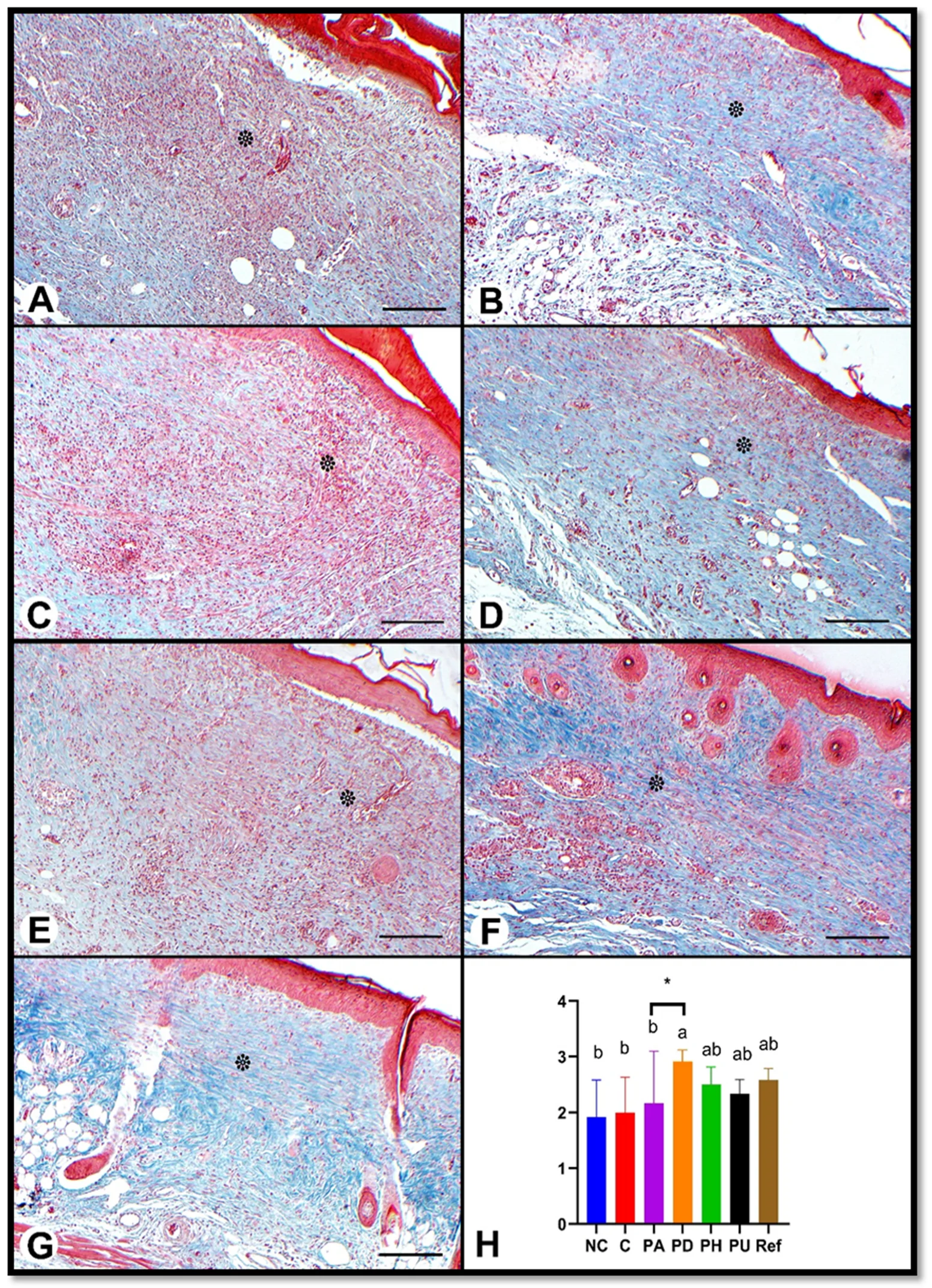
Histopathological evaluation of granulation tissue formation in the excisional wound model in mice treated with extracts of different *Prangos* species. Representative sections of wound tissues stained with Masson’s trichrome (MTC) are shown. (**A**) NC group: a small amount of granulation tissue with prominent cellular infiltration and capillaries in the wound area (asterisk). (**B**) C group: mature granulation tissue with limited cellular infiltration and few capillaries (asterisk). (**C**) PA group: granulation tissue with prominent cellular infiltration (asterisk). (**D**) PD group: mature granulation tissue in the wound area (asterisk). (**E**) PH group: moderately mature granulation tissue with prominent capillary formation (asterisk). (**F**) PU group: granulation tissue with prominent cellular infiltration and capillary formation (asterisk). (**G**) Ref group: mature granulation tissue in the wound area (asterisk). (**H**) Quantitative evaluation of granulation tissue formation in the control and experimental groups, * (*p* < 0.05), a,b: Different letters in the same row are statistically significant (*p* < 0.001). Masson’s trichrome staining, ×50 magnification; scale bar = 100 µm. NC: Negative control; C: Control (Vehicle ointment); PA: *Prangos abieticola;* PD: *Prangos denticulata;* PH: *Prangos heyniae;* PU: *Prangos uechtritzii*; Ref: Reference drug (nitrofuzaone).

The least granulation tissue development, which was significantly different from that of the PD group, occurred in the Control, NC, and PA groups (*p* ˂ 0.05). The most severe cell infiltration was observed in the NC group. A statistically significant difference in inflammatory infiltration was observed between the control, NC, PD, and Reference groups. Consequently, except for the PA group, the scores were similar in the other plant extract and reference groups (Figure 4A–G).

### 2.4. Immunohistochemical Analysis

The mean Type III collagen, SMA, and P4H score values for the control and experimental groups on day 5 of wound healing are summarized in Table 5.

**Figure 5:**
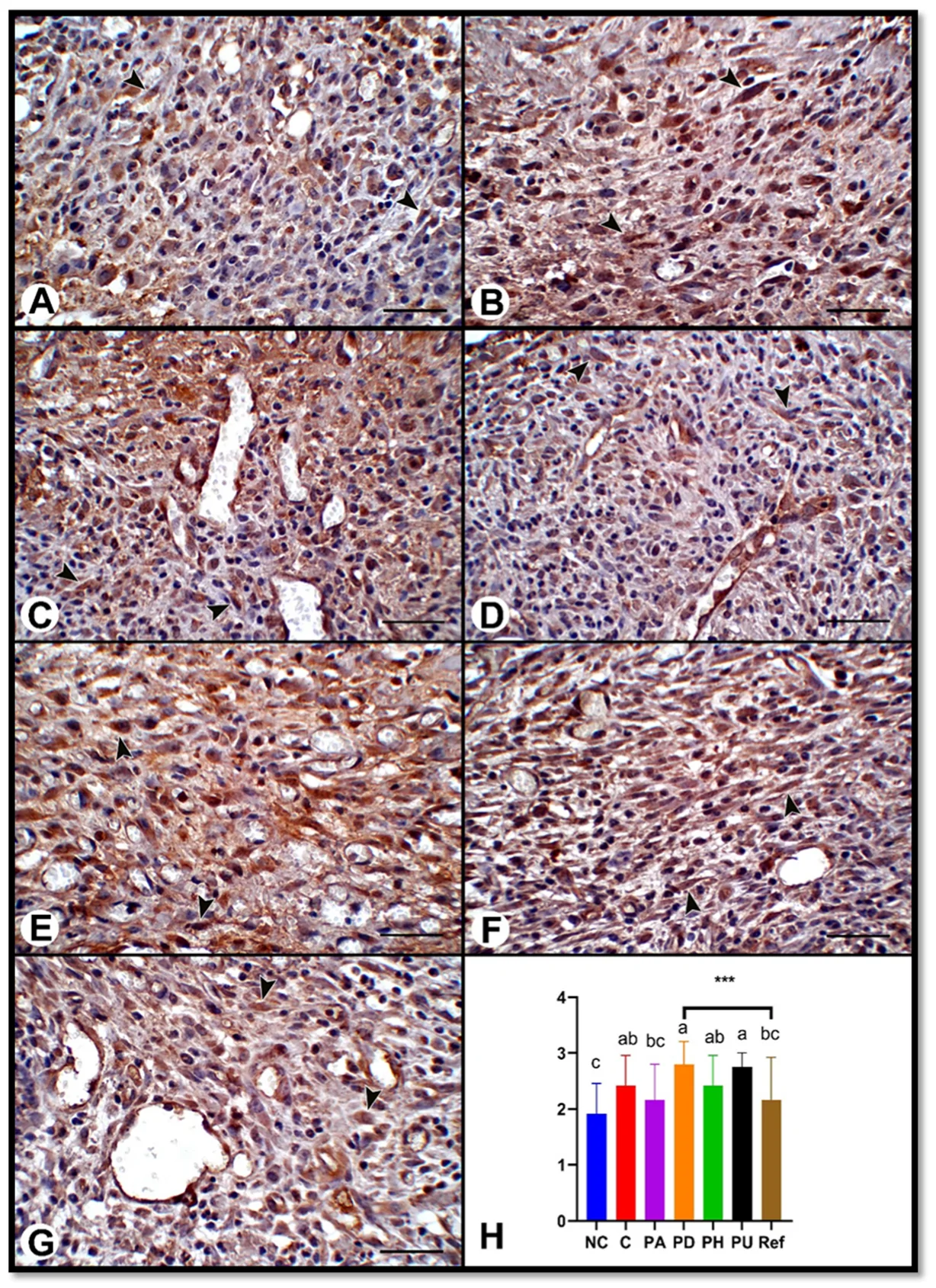
Immunohistochemical evaluation of Type III collagen expression in the excisional wound model in mice treated with extracts of different *Prangos* species. Representative images of Type III collagen immunopositivity are shown for the (**A**) NC group, (**B**) C group, (**C**) PA group, (**D**) PD group, (**E**) PH group, (**F**) PU group, and (**G**) Ref group. Arrowheads indicate Type III collagen-immunopositive cells. (**H**) Quantitative evaluation of Type III collagen immunopositivity in the control and experimental groups, *** (*p* < 0.001), a,b,c: Different letters in the same row are statistically significant (*p* < 0.001). Immunohistochemical staining, ×200 magnification; scale bar = 50 µm. NC: Negative control; C: Control (Vehicle ointment); PA: *Prangos abieticola;* PD: *Prangos denticulata;* PH: *Prangos heyniae;* PU: *Prangos uechtritzii*; Ref: Reference drug (nitrofuzaone).

Type III collagen immunostaining was prominent in fibroblasts, capillary endothelium, and adventitia. When groups were evaluated for collagen secretion, the least collagen positivity was observed in the NC, PA, and Reference groups, and the most in the PU and PD groups (*p* ≤ 0.001). Type III collagen secretion was similar between the control and PH groups (Figure 5A–G). No statistically significant differences were observed between the control, plant extract, and reference groups (*p* ≥ 0.05).

**Figure 6:**
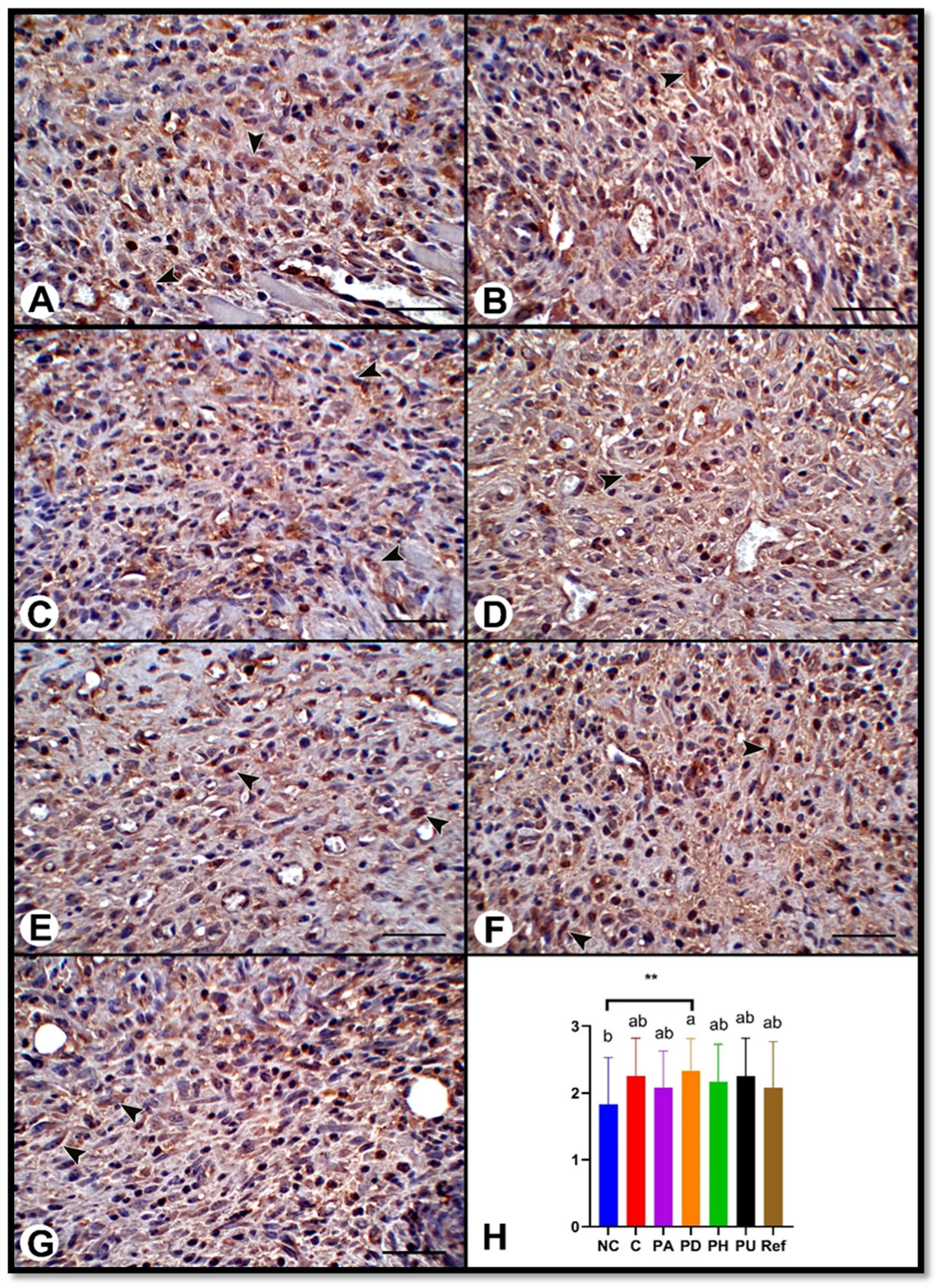
Immunohistochemical evaluation of α-smooth muscle actin (α-SMA) expression in the excisional wound model in mice treated with extracts of different *Prangos* species. Representative images of *α*-SMA immunopositivity are shown for the (**A**) NC group, (**B**) C group, (**C**) PA group, (**D**) PD group, (**E**) PH group, (**F**) PU group, and (**G**) Ref group. Arrowheads indicate α-SMA-immunopositive cells. (**H**) Quantitative evaluation of α-SMA immunopositivity in the control and experimental groups, ** (*p* < 0.01), a,b: Different letters in the same row are statistically significant (*p* < 0.001). Immunohistochemical staining, ×200 magnification; scale bar = 50 µm. NC: Negative control; C: Control (Vehicle ointment); PA: *Prangos abieticola;* PD: *Prangos denticulata;* PH: *Prangos heyniae;* PU: *Prangos uechtritzii*; Ref: Reference drug (nitrofuzaone).

SMA expression was detected in myofibroblasts and capillary endothelium in the wound area (Figure 6A–G). SMA immunostaining was least intense in the NC group. A statistically significant difference was observed only between the NC and PD groups (*p* ≤ 0.01). Similar intensity of SMA positivity was found between the control and other experimental groups (*p* ≥ 0.05).

**Figure 7:**
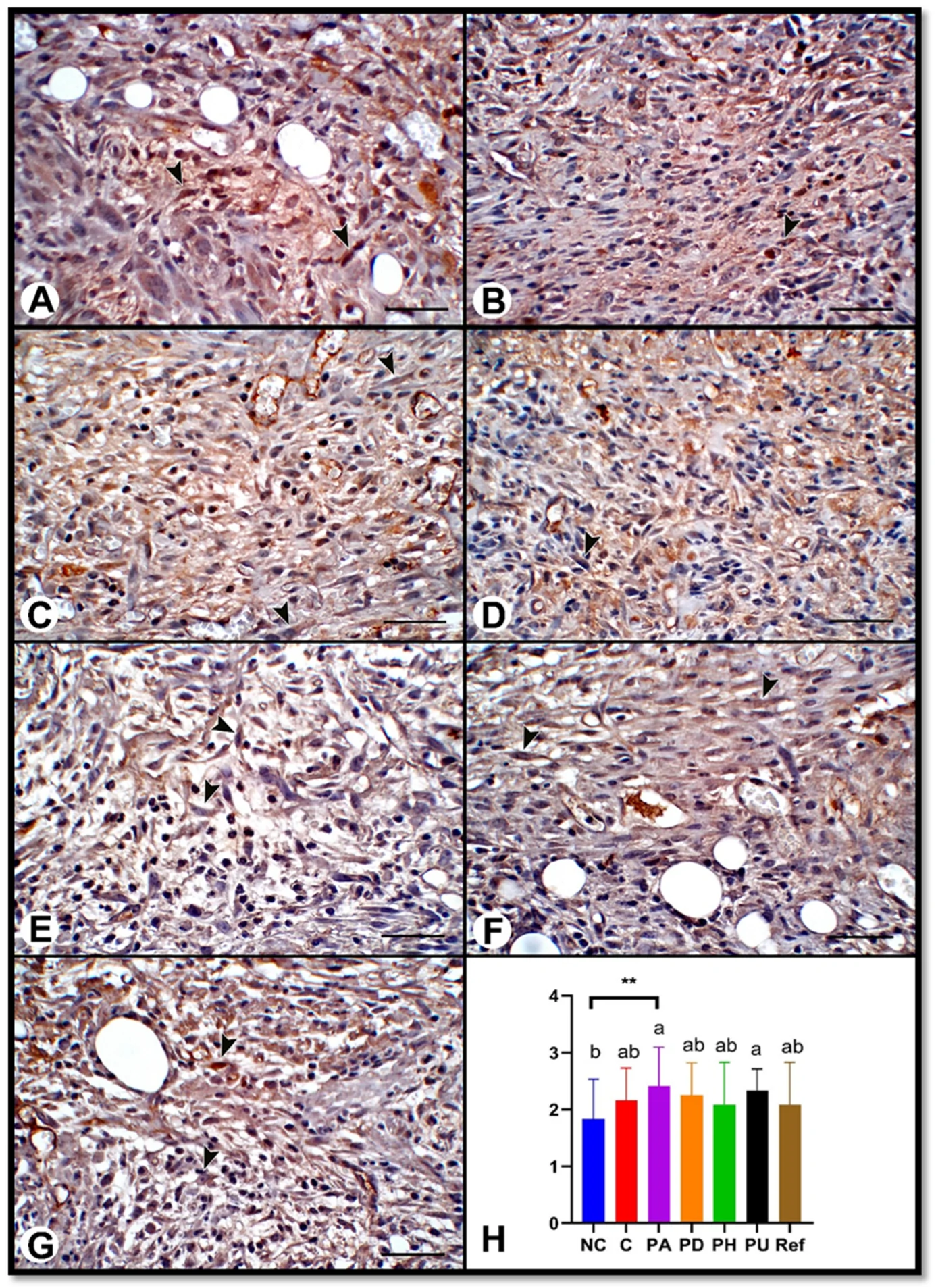
Immunohistochemical evaluation of prolyl 4-hydroxylase (P4H) expression in the excisional wound model in mice treated with extracts of different *Prangos* species. Representative images of P4H immunopositivity are shown for the (**A**) NC group, (**B**) C group, (**C**) PA group, (**D**) PD group, (**E**) PH group, (**F**) PU group, and (**G**) Ref group. Arrowheads indicate P4H-immunopositive cells. (**H**) Quantitative evaluation of P4H immunopositivity intensity in the control and experimental groups, ** (*p* < 0.01), a,b: Different letters in the same row are statistically significant (*p* < 0.001). Immunohistochemical staining, ×200 magnification; scale bar = 50 µm. NC: Negative control; C: Control (Vehicle ointment); PA: *Prangos abieticola;* PD: *Prangos denticulata;* PH: *Prangos heyniae;* PU: *Prangos uechtritzii*; Ref: Reference drug (nitrofuzaone).

P4H positivity was generally observed in the fibroblasts and capillary endothelium. The groups with the most prominent P4H immunostaining were the PA and PU groups, while the mildest positivity was found in the NC group (Figure 7A–G). A statistically significant difference in P4H staining intensity was observed only between the NC and other groups. No statistically significant difference was found between the plant extract and the reference group in terms of P4H immunopositivity.

## 3. Discussion

A wound is defined as a disruption of tissue integrity resulting from trauma, surgical procedures, or various pathological conditions and can be broadly classified as acute or chronic [32,33]. Following tissue injury, a coordinated series of cellular, physiological, and biochemical events is initiated to restore anatomical, physiological, and functional tissue integrity. The progression and outcome of wound healing can be influenced by several factors, including nutritional status, blood circulation, oxygenation, infection, hormonal status, smoking, and underlying chronic diseases [34,35].

The complexity of these processes has encouraged investigation of various therapeutic approaches for improving wound repair. Scientific evidence and clinical studies investigating traditional and complementary medicines for wound treatment have demonstrated considerable potential, with their effects being associated with anti-inflammatory activity as well as mechanisms such as enhanced collagen production and antimicrobial effects [36,37].

Given the multifaceted nature of wound healing, its assessment should incorporate complementary macroscopic, histological, and molecular parameters. Quantitative measurement of wound area provides an objective measure of wound closure and healing progression, whereas histopathological examination offers information on re-epithelialization, granulation tissue formation, inflammatory cell infiltration, collagen deposition, and tissue organization [38,39]. Accordingly, the combined evaluation of wound-area reduction, histopathological changes, and biochemical or molecular markers provides a more comprehensive assessment of the extent and quality of tissue repair [39]. Among the molecular regulators involved in this process, TGF-*β* plays an important role in fibroblast activity and extracellular matrix remodeling [40].

**Figure 8:**
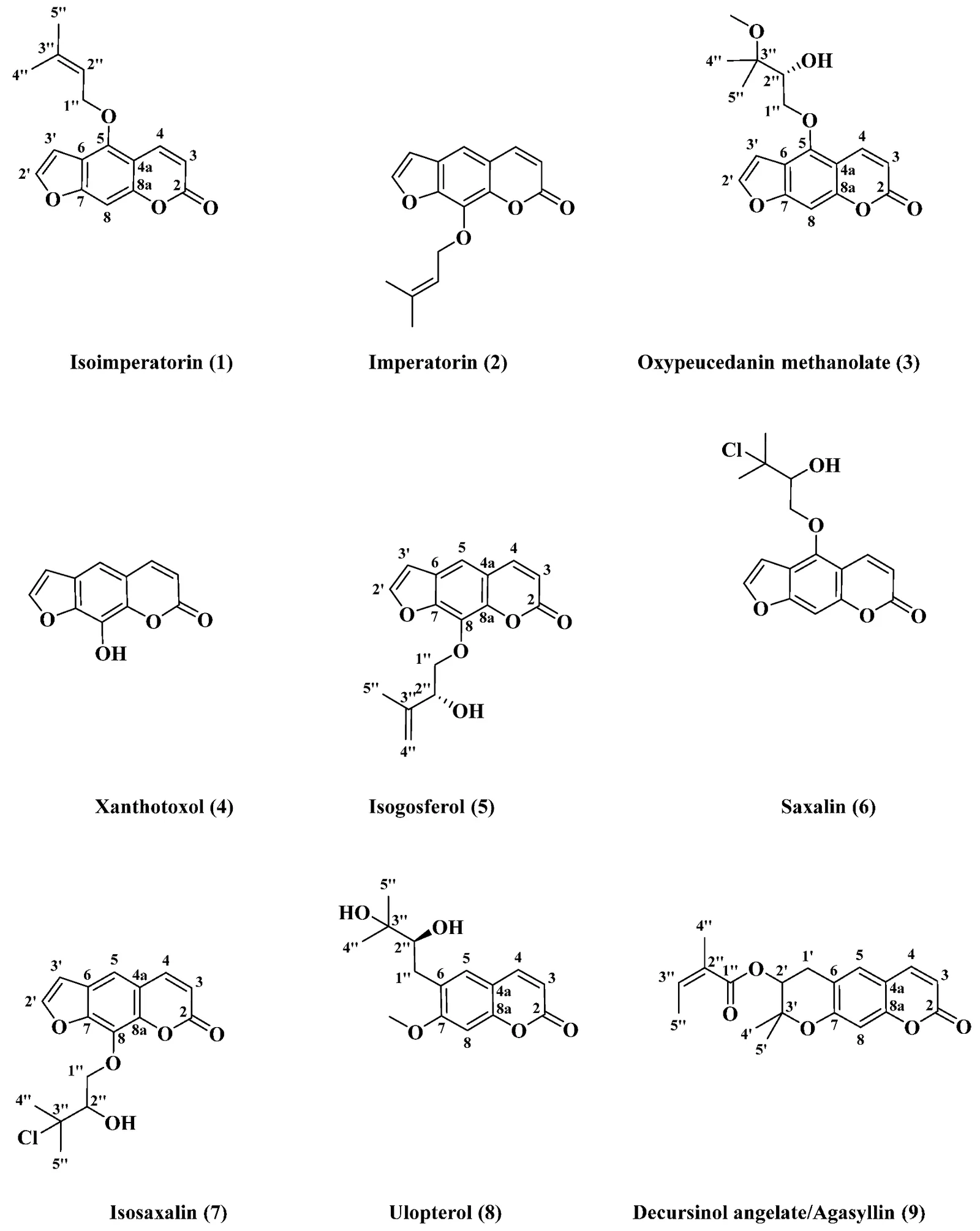
Coumarins isolated from *Prangos denticulata*.

In this study, we performed activity studies on endemic species of *Prangos* genus (*Prangos abieticola* Aytaç & H. Duman, *Prangos denticulata* Fisch. & C.A. Mey., *Prangos heyniae* H. Duman & M.F. Watson, and *Prangos uechtritzii* Boiss. & Hausskn.) to examine their wound-healing activity, as indicated in traditional medicine. These studies included wound area, histopathological, biochemical, and antioxidant analyses, and the results were compared with those of the reference and control groups. The wound-healing efficacy of these species was compared using a mouse wound model. Ointment formulations prepared using methanol extracts were created and applied to participants with excision-type wounds on their backs. The methanol extract of the aerial parts of *P. denticulata* showed better wound-healing activity compared to other species, and it was decided to conduct isolation studies on this species. Within the scope of phytochemical studies, larger quantities of the aerial parts of the active species, *P. denticulata*, were extracted using methanol to obtain phytochemicals. Chromatographic purification methods yielded furanocoumarins, such as isoimperatorin (**1**), imperatorin (**2**), oxypeucedanin methanolate (**3**), xanthotoxol (**4**), isogospherol (**5**), saxalin (**6**), isosaxalin (**7**), a simple coumarin, ulopterol (**8**), and a dihydropyranocoumarin (linear type), decursinol angelate (**9**) from the sub-fractions (Figure 8). The structures were elucidated using spectral analysis ^1^NMR, ^13^C NMR, and 2D NMR techniques. These compounds are frequently found in *Prangos* species, while others such as isogospherol, saxalin, isosaxaline, and decursinol angelate/agasyllin are relatively rarer coumarin-type compounds reported in the literature.

Isolation studies were aimed at and carried out on coumarins, as the dominant components of the plant. The coumarin-type compounds defined in the present study had been previously isolated from natural sources, mostly from plants. The structure elucidation of all furanocoumarins isolated from *P. denticulata*, isoimperatorin (**1**) [41,42,43,44]; imperatorin (**2**) [42,45]; oxypeucedanin methanolate (**3**) [46,47,48]; xanthotoxol (**4**) [49,50,51]; isogospherol (**5**) [52,53,54,55]; saxalin (**6**) [56,57,58,59]; isosaxalin (**7**) [60,61,62]; a simple coumarin, ulopterol (**8**) [63,64,65,66] and decursinol angelate/agasyllin (**9**) [67,68,69,70,71,72,73] were confirmed as comparing with the literature data and mass spectral data (including molecular and fragmentation ions) using LC–MS/MS analysis.

Coumarins are an important group of secondary metabolites classified among these phytochemical components, which are described in many sources for their wide variety of pharmacological effects [74], including antioxidant, antitumor, anti-HIV, antihypertensive, anticoagulant, antiviral, anti-inflammatory, analgesic, antidiabetic, antidepressant, etc. [75,76,77,78,79]. Studies suggest that although *Prangos* species contain a wide variety of constituents with diverse biological activities, coumarins are considered the major contributors to their biological effects because of their abundance in these species [9,10].

Coumarins have attracted considerable attention due to their potential anti-inflammatory and neuroprotective properties [80]. Naturally occurring coumarins and their derivatives have been extensively investigated for their ability to modulate inflammatory processes through multiple mechanisms, including antioxidant activity, suppression of pro-inflammatory cytokine production, and regulation of immune cell function [81].

Among furanocoumarins, imperatorin is a common furanocoumarin and has been the most extensively studied regarding biological activities. The experiments investigating its anti-inflammatory properties have been performed on LPS-activated RAW 264.7 cells and carrageenan-induced mouse paw edema. In general, it has been found to be caused by NOS and COX-2 inhibition. Isoimperatorin, an isomer of imperatorin, inhibits COX-dependent phases and TNF-*α*-induced expression and possesses anti-inflammatory properties [82]. In addition, imperatorin was also found in the literature review on its pharmacology, revealing that imperatorin has broad pharmacological activity and can be used for cancer prevention, neuroprotection, anti-inflammatory, antihypertensive, and antibacterial purposes [83]. Li et al. showed that imperatorin had promising effects in alleviating zymosan-induced pulmonary fibrosis, edema, and immune cell infiltration in mice, and concluded that imperatorin functions as an anti-inflammatory agent that reduces inflammation, swelling, and pulmonary fibrosis [84]. Isoimperatorin also promotes the healing of the intestinal mucosa by stimulating Treg cell formation, thus improving the symptoms of ulcerative colitis [85]. Furthermore, the pretreatment with isoimperatorin inhibits VEGF-induced endothelial cell proliferation, migration, and tube formation, and exhibits antiangiogenic effects [86]. Both of these furanocoumarins, isoimperatorin (**1**) and imperatorin (**2**), were isolated from *P. denticulata* and identified in this study.

Plants used in traditional medicine have long been utilized in various ways and have been the subject of research [87,88]. In addition, various medicinal plants have been used as remedies for skin ailments and have been included in traditional medicine practices since ancient times [89,90]. Studies on *Prangos* species have extensively described their phytochemical composition and activities, as well as their traditional uses, often used as wound-healing agents [9,10].

Considering that *Prangos* species are frequently used in traditional folk medicine for the treatment of burns and wounds, the presence of prenylated coumarins with antimicrobial activity strengthens the idea that *Prangos* species may play an important role in the aforementioned use in folk medicine. The dichloromethane extract of the roots of *Prangos hulusii*, an endemic species to Anatolia, isolated prenylated coumarins and furanocoumarins and reported their antimicrobial activities at concentrations between 5 and 125 g/mL [91]. The fact that similar plant species exhibit these effects is important for supporting the parallel effects.

Previous studies highlighting the wound-healing properties of *Prangos* species have also frequently investigated their essential oils and extracts. For example, treatment of fibroblast cells with essential oil from the roots of *P. ferulacea* (at concentrations of 1–32 μg/mL) significantly increased the migration rate of L929 cells (a mouse fibroblast cell line) in an MTT test, with migration rates of 87.05 ± 2.4% and 62.4 ± 0.09% observed at concentrations of 4 and 15 μg/mL, respectively (*p* < 0.001). Furthermore, these doses significantly increased fibroblast cell migration to the injured area, and the effect of this essential oil on collagen production was reported to significantly increase the total amount of type I collagen produced by L929 fibroblast cells in a collagen assay, in which type I collagen was used as an indicator (*p* < 0.001) [24].

A component called 3,5-nonadiene, obtained from *P. ferulacea* essential oil, also selectively inhibited endogenous NO release (in vitro) by rat peritoneal macrophages, with an IC_50_ value of 6.7 ± 0.8 μM, compared with the NOS inhibitor aminoguanidine (AG), which was used as a positive control [92]. In another study, the anti-inflammatory activity (70.98 ± 1.7%) of *P. platychlaena* fruit essential oil was investigated using a LOX inhibition assay, which is relevant to the prevention of inflammatory diseases, with nordihydroguaiaretic acid (NDGA) used as a positive control. Hexane (67.10 ± 2.5%) and methanol (50.11 ± 4.8%) fractions obtained from the essential oil also exhibited inhibitory activity [93]. In another experiment, the anti-inflammatory effects of two different extracts (methanolic and aqueous) obtained from *P. platychlaena* were examined at concentrations ranging from 10 to 1000 μg/mL. First, the cancer cells used in the experiment were stimulated with TNF-α, and the effects of the extracts on IL-8 and IL-6 release were investigated. IL-8 levels in CCL-221 cells decreased from 519.07 pg/mL to 28.3 pg/mL following treatment with the aqueous extract and to 92.73 pg/mL following treatment with the methanolic extract. Similarly, IL-6 levels decreased from 63 pg/mL to 1 pg/mL with the aqueous extract and to 4 pg/mL with the methanolic extract [94].

Prenylated coumarins obtained from the aerial parts of *P. haussknechtii* showed COX-1 enzyme inhibitory activity, with IC_50_ values of 36.8 and 47.7 μM, compared with aspirin, ibuprofen, and naproxen, which exhibited IC_50_ values of 600, 72.8, and 52.2 μM, respectively [95].

Furthermore, the furanocoumarins isolated from an *Angelica* species inhibit NO production in a dose-dependent manner. It was observed that treatment with these isolated compounds exerts their effects by suppressing the expression levels of iNOS, IL-1β, and COX-2 [71]. In addition, a previous study has determined that psoralen—which forms the core structure of linear furanocoumarins—accelerates bone fracture healing by activating osteoclast and osteoblast activities via the ERK signaling pathway. In short, psoralen has been identified as a potential agent that promotes bone fracture healing by activating osteoclasts and osteoblasts through the ERK signaling pathway [96].

Overall, the available scientific evidence provides support for the wound-healing and anti-inflammatory potential of *Prangos* species used in traditional medicine, although further studies are needed to clarify the underlying mechanisms and establish their therapeutic potential.

## 4. Limitations of the Study

This study has some limitations that should be considered when interpreting the findings. Although the assessment of TGF-*β*, hydroxyproline, TAS, TOS, and OSI provided complementary information on tissue repair, collagen deposition, and oxidative status, other important mechanisms involved in wound-healing, including inflammatory signaling, angiogenesis, re-epithelialization, extracellular matrix remodeling, and collagen organization, were not investigated in detail. Although nine coumarin-type constituents were isolated and structurally characterized from *P. denticulata*, the present study did not establish which individual compound(s), or whether synergistic interactions among constituents, are responsible for the observed wound-healing activity. Thus, the association between the phytochemical composition of *P. denticulata* and its biological activity remains to be further clarified through activity-guided isolation and evaluation of the isolated compounds individually and in combination. Finally, further studies addressing topical tissue exposure, safety, dose–response relationships, and longer-term wound remodeling are warranted before the findings can be extrapolated to clinical applications.

## 5. Materials and Methods

### 5.1. Plant Materials

Plant samples were collected from different localities in Anatolia. Taxonomic identification of the *Prangos* species was confirmed by Prof. Hayri Duman from the Department of Biological Sciences, Faculty of Art and Sciences, Gazi University (Ankara-Türkiye). (*Prangos abieticola* Aytaç & H.Duman, *Prangos denticulata* Fisch. & C.A.Mey., *Prangos heyniae* H.Duman & M.F.Watson, and *Prangos uechtritzii* Boiss. & Hausskn.) were collected during flowering period (Table 6). The plant’s scientific names have been checked with https://powo.science.kew.org (accessed on 8 October 2026) [97]. The material was dried in the shade in a cool place, then powdered (ground in a pulverizing mill) under suitable conditions, separating the aerial parts and roots. Voucher specimens were deposited in the Ankara University Faculty of Pharmacy Herbarium (AEF). The collected localities and dates of the plant samples, along with the herbarium numbers, are listed in Table 6.

### 5.2. Nuclear Magnetic Resonance (NMR) and Liquid Chromatography—Tandem Mass Spectrometry (LC-MS/MS) Analysis

1D and 2D NMR spectra were obtained on an NMR spectrometer for spectral analyses using deuterated solvents with tetramethylsilane (TMS) as an internal standard in 5 mm NMR tubes. In addition to ^1^H-NMR, ^13^C NMR, COSY, HSQC, and HMBC analyses were also used to elucidate the structures of the compounds. Chemical shifts are expressed in ppm with coupling constants (*J*) in Hertz (Hz). NMR analyses were performed using a Bruker Avance Neo 500 MHz (Germany) instrument located in the central laboratory of the Faculty of Pharmacy, Ankara University. Silica gel (70–230 and 230–400 mesh) for column chromatography and silica gel 60 F_254_ TLC plates for thin-layer chromatography was procured from Merck. The often-used solvents, such as ethyl acetate, methanol, and *n*-hexane, were purchased from Isolab and Merck companies.

In addition, liquid chromatography-tandem mass spectrometry (LC-MS/MS) analyses were performed as a purchased analytical service to confirm or verify the structures in the Hacettepe University Advanced Technologies Application and Research Center Laboratories (HÜNİTEK). The LC-MS/MS analyses were performed on a Bruker maXis II ETD instrument (Bruker Daltonics, Bremen, Germany), which combines a Quadrupole (Q) analyzer for selecting the desired ions with a time-of-flight (TOF) analyzer for high-precision mass measurement. Samples dissolved in acetonitrile were introduced into the system by direct injection using a syringe pump operating at a flow rate of 3 µL/min. An electrospray ionization (ESI) source operating in positive ion mode was used in the analyses, with capillary voltage set at 3500 V, nebulizer gas pressure at 0.3 bar, drying gas flow rate at 4 L/min, and drying gas temperature at 200 °C. Nitrogen gas was used as the nebulization and drying gas. Mass calibration was performed using sodium format adducts, and mass spectra were obtained in full scan mode in the *m*/*z* 50–3000 range. Additionally, MS/MS analyses were performed using collision-induced decomposition (CID) where necessary. Bruker Compass Data Analysis software was used for data collection and evaluation.

### 5.3. Extraction of the Plant Materials for Biological Activity Screening

The crude extracts from powdered plant materials (10 g each) prepared with methanol (100 mL) at room temperature using Soxhlet extraction in multiple extraction cycles (at least 10 times) were used for the activity screening tests, as described in a previous study [79]. However, *P. uechtritzii* (PU) extract was prepared using the same extraction method in this study. After the extraction process, all extracts were filtered, and the solvent was removed under low pressure at 30 °C and stored in the refrigerator.

### 5.4. Extraction of the Plant Material for the Isolation Studies

Powdered plant material (500 g) (the aerial parts of *P. denticulata* were found to be active in the analysis tests) was macerated with methanol in a dark, cool place for a short period. The first extract was filtered, and then the remaining parts of the plant were extracted using the Soxhlet apparatus (9 L of 100% methanol with 1 L extractor volume and 2 L flask volume, each cycle 40 min, and at least 10 siphons were performed). The solvent was evaporated at low pressure, in a Rotavapor R-100 device at a rotation speed of 65 rpm, at less than 30 °C.

### 5.5. Isolation Process of the Plant Phytochemicals

A portion of the crude extract (80 g) was subjected to vacuum-assisted column chromatography over silica gel and eluted roughly and rapidly with hexane, ethyl acetate, and methanol to yield eight major fractions (Fr. 1–8). Fraction analysis was performed based on thin-layer chromatography (TLC) on silica gel 60 F_254_ plates developed with various hexane–ethyl acetate solvent systems. Final purification of the constituents was achieved by recrystallization, repeated column chromatography, and preparative TLC, as appropriate to obtain nine known coumarins.

### 5.6. Biological Activity Methods

#### 5.6.1. Wound-Healing Activity

Biological activity studies were conducted to investigate and observe the wound-healing effects of plant extracts in vivo in mice. A circular excision wound model was used for this purpose. Approval for all procedures to be performed on animals in this study was granted by the Gazi University Local Ethics Committee for Animal Experiments with approval number G.U.E.T-21.072, and the experiments were conducted in accordance with the principles of the ethics committee.

#### 5.6.2. Experimental Animals

In the experiments, 84 healthy male Swiss albino mice, 10–12 weeks old (25–30 g), were used. Throughout the experiment, the animals were housed at 21–24 °C and 45–55% relative humidity under a 12 h light/12 h dark cycle and provided standard pellet feed and water *ad libitum* [98,99]. In the experiments, 84 healthy male Swiss albino mice, aged 10–12 weeks and weighing 25–30 g, were used. Throughout the experiment, the animals were housed at 21–24 °C and 45–55% relative humidity under a 12 h light/12 h dark cycle and provided standard pellet feed and water *ad libitum* [98,99]. The 84 mice were randomly divided into 14 groups of six animals each (n = 6). The groups were as follows: NC1 and NC2, wound-induced non-treated groups; C1 and C2, wound-induced vehicle-treated groups receiving the ointment base; PA1 and PA2, wound-induced groups treated with a simple ointment containing 5% methanolic extract of *P. abieticola*; PD1 and PD2, wound-induced groups treated with a simple ointment containing 5% methanolic extract of *P. denticulata*; PH1 and PH2, wound-induced groups treated with a simple ointment containing 5% methanolic extract of *P. heyniae*; PU1 and PU2, wound-induced groups treated with a simple ointment containing 5% methanolic extract of *P. uechtritzii*; and Ref1 and Ref2, wound-induced groups treated with a simple ointment containing 0.2% nitrofurazone. The simple ointment base was prepared by mixing anhydrous lanolin (5%, *w*/*w*), solid paraffin (5%, *w*/*w*), cetostearyl alcohol (5%, *w*/*w*), and petrolatum (85%, *w*/*w*) until a homogeneous preparation was obtained. The experimental samples were applied externally to the wounds at a dose of 0.1 g per day. The preparations were applied topically and directly to the excision wounds as a thin, uniform layer using a sterile applicator, with care taken to cover the entire wound surface without causing additional mechanical irritation. The treatment was performed once daily throughout the experimental period. The animals were housed individually in clean cages, which were regularly cleaned throughout the experimental period.

Animals in groups NC1, C1, PA1, PD1, PH1, PU1, and Ref1 were sacrificed on day 5 for immunohistochemical (IHC) and TGF-*β* assessments, whereas animals in groups NC2, C2, PA2, PD2, PH2, PU2, and Ref2 were sacrificed on day 10 for routine histopathological analysis and measurement of hydroxyproline, total antioxidant status (TAS), and total oxidant status (TOS) levels (Table 7).

#### 5.6.3. Excision Wound Model

After intraperitoneal administration of 5 mg/kg xylazine and 100 mg/kg ketamine for general anesthesia, the dorsal area was clipped, and the skin was cleansed with 70% isopropyl alcohol followed by 0.2% chlorhexidine solution immediately before wound creation to prepare the surgical field under aseptic conditions. Two circular excision wounds with a diameter of 6 mm were created in the dorsal region [100]. The treatment protocol was applied to the wounds once daily throughout the 10-day experimental period, until the predefined experimental endpoint (Table 7), and the wounds were photographed every other day (0, 2, 4, 6, 8, and 10 days) [98,101]. The Bersoft Image Measurement program, which provides area measurements, was used to calculate the reduction in wound area [102].

#### 5.6.4. Termination of the Experimental Procedure

On the 5th day of the experiment, the animals were sacrificed to perform immunohistochemical analyses and *TGF-β* level measurements. On the 10th day, wound tissue samples were collected following sacrifice. Tissue samples for routine histopathological examination were excised from the wound area together with a 1 cm margin of surrounding healthy skin. The other sample was used to determine hydroxyproline, total antioxidant status, and total oxidant status levels.

#### 5.6.5. Histopathological Analyses

At the end of the experiment, skin samples were collected from all groups and fixed in 10% buffered formaldehyde. After washing with tap water, paraffin blocks were prepared from the tissues that underwent routine procedures, and 5 µm thick sections were taken with a microtome, stained with Hematoxylin-Eosin (HE) and Masson’s Trichrome (MT) methods, and examined under a light microscope [103]. The tissues were qualitatively evaluated for ulceration, re-epithelialization, fibroblast proliferation, mono/polymorphonuclear cells, angiogenesis, and collagen proliferation to determine the phase of wound-healing (Table 8).

#### 5.6.6. Determination of Type III Collagen, α-Smooth Muscle Actin, and Prolyl-4-hydroxylase Levels in Tissues by Immunohistochemical (IHC) Analysis

The avidin-biotin-peroxidase method was used for immunohistochemical analyses. 4-micron-thick sections taken from paraffin blocks were deparaffinized with xylene and then dewatered by passing them through a graduated alcohol series. Antigen retrieval was performed via heat-induced epitope retrieval by boiling tissue sections in 0.01 M sodium citrate buffer (pH 6.0) in a microwave oven at 95–98 °C for 20 min, followed by cooling at room temperature. Then the sections were washed with PBS and incubated in 3% hydrogen peroxide prepared with methyl alcohol for 10 min to inhibit endogenous peroxidase activity. To prevent non-specific binding, the sections were incubated with 1% normal goat serum for 1 h. According to the manufacturer’s procedure, anti-type III collagen (Col III), *α*-smooth muscle actin (*α*-SMA), and prolyl-4-hydroxylase (P4H) primary antibodies diluted in PBS were incubated overnight at 4 °C. The sections were washed again with PBS and treated with biotinylated secondary antibody droplets for 10 min. Subsequently, the tissues were washed with PBS and treated with streptavidin peroxidase for 10 min. 3,3′-Diaminobenzidine (DAB) was used as a color-releasing substrate. After applying these solutions to the sections, the reaction was stopped as soon as a color change occurred. In the final step, the sections were stained with Mayer’s Hematoxylin for 2 min, washed with tap water, passed through an alcohol-xylene series, coated with Entellan, and evaluated by light microscopy at appropriate magnification. A semi-quantitative evaluation was performed on the immunohistochemical staining. In each animal section, Col III, SMA, and prolyl-4-hydroxylase immunopositivity in the wound-line region were scored as follows: no staining: 0, weak staining: 1, moderate staining: 2, and strong staining: 3, and statistical comparisons were made between groups [104].

### 5.7. Biochemical Analyses

Determination of TGF-*β* Levels: Tissues, removed 5 days after wound creation, were homogenized, and protein extraction was performed using T-PER reagent. Protein concentrations were determined according to the manufacturer’s instructions for the Tissue Protein Extraction Reagent, and TGF-*β* levels in the samples were determined according to the manufacturer’s instructions for an enzyme-linked immunosorbent assay (ELISA) kit (E-EL-R0162; Elabscience Biotechnology Co., Ltd., Wuhan, China) [105].

Determination of Hydroxyproline Levels in Tissues: Tissue samples (5 mg) were collected, homogenized with 100 µL of water, and 100 µL of 12 M HCl was added to the homogenized tissues. The samples were placed in tightly sealed vials and hydrolyzed at 120 °C for 3 h. The resulting supernatant (20 µL) was placed in a 96-well plate and dried at 60 °C. Samples were measured using a hydroxyproline assay kit (E-BC-K061-S; Elabscience Biotechnology Co., Ltd., Wuhan, China) with a microplate reader at a wavelength of 560 nm [106].

Determination of Total Antioxidant (TAS), Total Oxidant (TOS) Levels, and Oxidative Stress Index (OSI) in Tissue: Antioxidant activity is an important parameter in wound-healing. Therefore, the oxidative stress index in treated tissues is an important parameter. Tissue TAS and TOS levels were evaluated using commercial kits (Rel Assay, Mega Tıp San. Tic. Ltd., Şti., Gaziantep, Türkiye). For the TAS level, the scavenging effects of tissue antioxidants on the ABTS [2,2′-azino-bis (3-ethylbenzothiazoline-6-sulfonic acid) radical cation] were evaluated. For the TOS level, the capacity of tissue oxidants in the samples to oxidize ferrous ion-O-dianizine to ferric ions was measured. The OSI was calculated using these parameters [107]. The OSI was calculated as the ratio of the total oxidant status (TOS) to the total antioxidant status (TAS), with TAS values converted to micromoles. Accordingly, the OSI (arbitrary unit) was determined using the following equation: OSI = TOS (μmol H_2_O_2_ equivalent/L)/TAS (μmol Trolox equivalent/L) × 100.

### 5.8. Statistical Analyses

The obtained data were expressed as mean ± standard error of the mean (SEM). A *p*-value of <0.05 was considered statistically significant. Data analysis was performed using appropriate statistical methods with the GraphPad Prism 7 software. The data obtained from the histological and immunohistochemical scores were expressed as the mean and standard error. Statistical significance was set at *p* < 0.05. The Shapiro–Wilk normality test was applied to determine whether the raw values of all measured parameters showed a normal distribution. If the variances in the groups were homogeneous, one-way analysis of variance (ANOVA) and post hoc Tukey tests were used for pairwise comparisons.

## 6. Conclusions

In this study, the methanol extracts of four different endemic species of the genus *Prangos* (*P. abieticola* Aytaç & H.Duman, *P. denticulata* Fisch. & C.A. Mey., *P. heyniae* H. Duman & M.F. Watson, and *P. uechtritzii* Boiss. & Hausskn.) were evaluated for the wound-healing potentials, and *P. denticulata* was determined to be the most active species with wound-healing properties based on the wound area, biochemical analyses, antioxidant studies, and histopathological findings.

It has been concluded that the methanol extract of *P. denticulata*, which has been evaluated for its wound-healing properties, may hold significant potential for the development of natural products regarding wound-healing. However, the isolated compounds should also be examined in this context. Although coumarin and its derivatives are known to exhibit these effects, synergistic effects may also occur in the extracts. Therefore, further research is required. It is necessary to investigate which substance or group of substances caused the relatively higher wound-healing activity shown by the methanol extract. In addition, the planning of different activity methods to explain the mechanism of action underlying this effect will be included in future studies. Since predominantly higher amounts of furanocoumarin compounds were isolated from *P. denticulata*, it can be assumed that the activity may be attributed to these compounds.

In conclusion, this endemic species, *P. denticulata*, found in the flora of Türkiye, has been examined in terms of its effects and phytochemical components in this study. Furthermore, these studies can be supported and developed with different research in the future.

## Figures and Tables

**Table 1:** The measurements of the wound area (mm^2^).

Groups	Day 2	Day 4	Day 6	Day 8	Day 10
NC	22.338 ± 1.823	30.879 ± 4.013	29.434 ± 3.911	12.045 ± 1.321	6.406 ± 1.478
C	37.668 ± 4.877	45.114 ± 10.807	29.565 ± 3.603	15.878 ± 3.088	9.724 ± 1.574
PA	32.303 ± 2.413	37.778 ± 5.277	24.966 ± 1.787	7.227 ± 2.205 *	3.833 ± 1.239 *
PD	24.090 ± 1.426	28.799 ± 3.821	16.863 ± 2.932	6.242 ± 1.736 *	1.752 ± 0.139 **
PH	29.784 ± 2.917	39.092 ± 7.710	28.251 ± 8.288	11.695 ± 2.0818	4.161 ± 1.744
PU	33.342 ± 3.662	32.740 ± 2.891	18.725 ± 3.459	7.227 ± 0.814 *	1.862 ± 1.118 **
Ref	28.514 ± 4.569	22.995 ± 1.454	16.425 ± 1.229	5.519 ± 1.403 *	1.971 ± 0.599 **

Results were compared to the control group. *: *p* < 0.05; **: *p* < 0.01; NC: Negative control; C: Control (Vehicle ointment); PA: *Prangos abieticola;* PD: *Prangos denticulata;* PH: *Prangos heyniae;* PU: *Prangos uechtritzii*; Ref: Reference drug (nitrofuzaone).

**Table 2:** TGF-*β* values (Day 5).

Groups	TGF-*β* (µg/mg)
NC	0.5900 ± 0.0871
C	0.7540 ± 0.0877
PA	1.8133 ± 0.5373 *
PD	1.4075 ± 0.1461
PH	1.0720 ± 0.0628
PU	0.9640 ± 0.1271
Ref	1.1275 ± 0.1676

Results were compared to the control group. *: *p* < 0.05; NC: Negative control; C: Control (Vehicle ointment); PA: *Prangos abieticola;* PD: *Prangos denticulata;* PH: *Prangos heyniae;* PU: *Prangos uechtritzii*; Ref: Reference drug (nitrofuzaone).

**Table 3:** TAS-TOS and OSI values (Day 10).

Groups	Hydroxyproline Amount (µg/mg)	TAS (mmol/L)	TOS (μmol/L)	OSI (AU)
NC	2.4895 ± 0.0783	1.3367 ± 0.0695	6.4220 ± 0.7509	0.480
C	1.8830 ± 0.1514	1.5675 ± 0.1487	4.6533 ± 1.6334	0.297
PA	2.3322 ± 0.3763	1.5060 ± 0.1212	3.1200 ± 0.6976	0.207
PD	2.1903 ± 0.1204	1.7580 ± 0.0472 *	2.8625 ± 0.5738	0.162
PH	3.1990 ± 0.1289 *	1.5850 ± 0.0912	4.6320 ± 1.025	0.292
PU	2.5505 ± 0.3297	1.6520 ± 0.07486	3.1740 ± 0.3926	0.192
Ref	2.3834 ± 0.1114	1.7750 ± 0.1372 *	3.4550 ± 0.8749	0.195

Results were compared to the control group. *: *p* < 0.05; Total Antioxidant (TAS), Total Oxidant (TOS) Levels, and Oxidative Stress Index (OSI); NC: Negative control; C: Control (Vehicle ointment); PA: *Prangos abieticola;* PD: *Prangos denticulata;* PH: *Prangos heyniae;* PU: *Prangos uechtritzii*; Ref: Reference drug (nitrofuzaone).

**Table 4:** Average wound healing score values according to microscopic examination in the control and experimental groups.

	NC	C	PA	PD	PH	PU	Ref	SEM	*p*
Re-epithelialization	2.17 ± 0.31	2.17 ± 0.17	2.08 ± 0.35	2.67 ± 0.17	2.33 ± 0.11	2.50 ± 0.13	2.50 ± 0.13	0.08	ns
Granulation Tissue	1.92 ± 0.27 ^b^	2.00 ± 0.26 ^b^	2.17 ± 0.38 ^b^	2.92 ± 0.08 ^a^	2.50 ± 0.13 ^ab^	2.33 ± 0.11 ^ab^	2.58 ± 0.08 ^ab^	0.09	0.05
Collagen deposition	1.83 ± 0.33	2.17 ± 0.31	2.33 ± 0.36	2.83 ± 0.11	2.42 ± 0.15	2.42 ± 0.33	2.75 ± 0.11	0.10	ns
Inflammatory Cells	2.25 ± 0.36 ^a^	1.33 ± 0.21 ^b^	1.83 ± 0.40 ^ab^	1.00 ± 0.22 ^b^	1.75 ± 0.21 ^ab^	1.75 ± 0.36 ^ab^	1.17 ± 0.11 ^b^	0.12	0.05
Angiogenesis	2.83 ± 0.17	2.50 ± 0.34	2.42 ± 0.27	1.83 ± 0.40	2.58 ± 0.20	2.67 ± 0.21	2.58 ± 0.15	0.10	ns
Ulcer	2.50 ± 0.22	3.00 ± 0.00	2.25 ± 0.40	3.00 ± 0.00	3.00 ± 0.00	2.33 ± 0.49	3.00 ± 0.00	0.10	ns

^a,b^: Different letters in the same row are statistically significant (*p* < 0.001); ns: The difference is not statistically significant (*p* > 0.05); NC: Negative control; C: Control (Vehicle ointment); PA: *Prangos abieticola;* PD: *Prangos denticulata;* PH: *Prangos heyniae;* PU: *Prangos uechtritzii*; Ref: Reference drug (nitrofuzaone); SEM: Standard Error of the Mean.

**Table 5:** Mean scores for Type III collagen, SMA, and P4H release intensity in the wound area in the control and experimental groups.

	NC	C	PA	PD	PH	PU	Ref	SEM	*p*
Type III Collagen	1.92 ± 0.10 ^c^	2.42 ± 0.10 ^ab^	2.17 ± 0.12 ^bc^	2.72 ± 0.09 ^a^	2.42 ± 0.10 ^ab^	2.75 ± 0.05 ^a^	2.17 ± 0.14 ^bc^	0.04	0.001
SMA	1.83 ± 0.13 ^b^	2.25 ± 0.10 ^ab^	2.08 ± 0.10 ^ab^	2.33 ± 0.09 ^a^	2.17 ± 0.10 ^ab^	2.25 ± 0.10 ^ab^	2.08 ± 0.13 ^ab^	0.04	0.01
P4H	1.83 ± 0.13 ^b^	2.17 ± 0.10 ^ab^	2.42 ± 0.13 ^a^	2.25 ± 0.10 ^ab^	2.08 ± 0.14 ^ab^	2.33 ± 0.07 ^a^	2.08 ± 0.14 ^ab^	0.05	0.01

^a,b,c^: Different letters in the same row are statistically significant (*p* < 0.001); NC: Negative control; C: Control (Vehicle ointment); PA: *Prangos abieticola;* PD: *Prangos denticulata;* PH: *Prangos heyniae;* PU: *Prangos uechtritzii*; Ref: Reference drug (nitrofuzaone); SEM: Standard Error of the Mean.

**Table 6:** Collection localities, dates, and herbarium voucher numbers of the *Prangos* species.

Species	Locality	Date	Herbarium No
*Prangos abieticola* Aytaç & H.Duman	Konya-Akseki road, around the stone area	19 June 2019	AEF 28893
*Prangos denticulata* Fisch. & C.A.Mey	Ankara-Mamak, in a hilly area	10 June 2019	AEF 28894
*Prangos heyniae* H.Duman & M.F.Watson	Konya, Bozkır, around, in the stone area	12 June 2019	AEF 28892
*Prangos uechtritzii* Boiss. & Hausskn.	Kayseri Yahyalı district	30 June 2020	AEF 28896

**Table 7:** Groups of experimental animals.

Groups	Treatment	Termination
NC_1_ (n = 6)	Wound-induced, non-treated group	Sacrificed on day 5 for IHC and TGF-*β* assessment.
NC_2_ (n = 6)		Sacrificed on day 10 for routine histopathological analysis and measurement of hydroxyproline, TAS, and TOS levels.
C_1_ (n = 6)	Wound-induced, vehicle (ointment base)-treated group	Sacrificed on day 5 for IHC and TGF-*β* assessment.
C_2_ (n = 6)		Sacrificed on day 10 for routine histopathological analysis and measurement of hydroxyproline, TAS, and TOS levels.
PA_1_ (n = 6)	Wound-induced, simple ointment containing 5% methanolic extract of *Prangos abieticola*-treated group	Sacrificed on day 5 for IHC and TGF-*β* assessment.
PA_2_ (n = 6)		Sacrificed on day 10 for routine histopathological analysis and measurement of hydroxyproline, TAS, and TOS levels.
PD_1_ (n = 6)	Wound-induced, simple ointment containing 5% methanolic extract of *Prangos denticulata*-treated group.	Sacrificed on day 5 for IHC and TGF-β assessment.
PD_2_ (n = 6)		Sacrificed on day 10 for routine histopathological analysis and measurement of hydroxyproline, TAS, and TOS levels.
PH_1_ (n = 6)	Wound-induced, simple ointment containing 5% methanolic extract of *Prangos heyniae*-treated group.	Sacrificed on day 5 for IHC and TGF-*β* assessment.
PH_2_ (n = 6)		Sacrificed on day 10 for routine histopathological analysis and measurement of hydroxyproline, TAS, and TOS levels.
PU_1_ (n = 6)	Wound-induced, simple ointment containing 5% methanolic extract of *Prangos uechtritzii*-treated group.	Sacrificed on day 5 for IHC and TGF-*β* assessment.
PU_2_ (n = 6)		Sacrificed on day 10 for routine histopathological analysis and measurement of hydroxyproline, TAS, and TOS levels.
Ref_1_ (n = 6)	Wound-induced, simple ointment containing 0.2% nitrofurazone-treated group	Sacrificed on day 5 for IHC and TGF-*β* assessment.
Ref_2_ (n = 6)		Sacrificed on day 10 for routine histopathological analysis and measurement of hydroxyproline, TAS, and TOS levels.

IHC: Immuno-histochemical; TGF-*β*: Transforming growth factor-*β*; TAS: Total antioxidant; TOS: Total oxidant; NC: Negative control; C: Control (Vehicle ointment); PA: *Prangos abieticola;* PD: *Prangos denticulata;* PH: *Prangos heyniae;* PU: *Prangos uechtritzii*; Ref: Reference drug (nitrofuzaone).

**Table 8:** Histopathological scoring system for re-epithelialization, granulation tissue, collagen deposition, inflammatory cell infiltration, angiogenesis, and ulceration.

Score	Re-Epithelialization	Granulation Tissue	Collagen Deposition	Inflammatory Cell	Angiogenesis	Ulcer
0	None	None or immature	None	None	None	Large or deep ulcer, abscess formation
1	Partial	Few	Few	Few	Less than 5 vessels	Large ulcer
2	Completed but immature or thin	Moderate maturation	Moderate	Moderate	6–10 vessels	None or very small
3	Completed and mature	Mature	Abundant	Abundant	More than 10 vessels	None

## Data Availability

Data are contained within the article.
